# Food-grade argan oil supplementation in molasses enhances fermentative performance and antioxidant defenses of active dry wine yeast

**DOI:** 10.1186/s13568-015-0159-7

**Published:** 2015-12-01

**Authors:** Esther Gamero-Sandemetrio, Max Torrellas, María Teresa Rábena, Rocío Gómez-Pastor, Agustín Aranda, Emilia Matallana

**Affiliations:** Departament de Bioquímica i Biologia Molecular, Universitat de València, València, Spain; Departamento de Biotecnología, Instituto de Agroquímica y Tecnología de Alimentos, CSIC, Avda Agustín Escardino, 7. Paterna, 46980 Valencia, Spain; Departament d’Estadística i Investigació Operativa, Universitat de València, Valencia, Spain

**Keywords:** Active dry wine yeasts, Food-grade argan oil, antioxidant capacity, Oxidative damage, Antioxidant defenses

## Abstract

The tolerance of the yeast *Saccharomyces cerevisiae* to desiccation is important for the use of this microorganism in the wine industry, since active dry yeast (ADY) is routinely used as starter for must fermentations. Both biomass propagation and dehydration cause cellular oxidative stress, therefore negatively affecting yeast performance. Protective treatments against oxidative damage, such as natural antioxidants, may have important biotechnological implications. In this study we analysed the antioxidant capacity of pure chemical compounds (quercetin, ascorbic acid, caffeic acid, oleic acid, and glutathione) added to molasses during biomass propagation, and we determine several oxidative damage/response parameters (lipid peroxidation, protein carbonylation, protective metabolites and enzymatic activities) to assess their molecular effects. Supplementation with ascorbic, caffeic or oleic acids diminished the oxidative damage associated to ADY production. Based on these results, we tested supplementation of molasses with argan oil, a natural food-grade ingredient rich in these three antioxidants, and we showed that it improved both biomass yield and fermentative performance of ADY. Therefore, we propose the use of natural, food-grade antioxidant ingredients, such as argan oil, in industrial processes involving high cellular oxidative stress, such as the biotechnological production of the dry starter.

**Electronic supplementary material:**

The online version of this article (doi:10.1186/s13568-015-0159-7) contains supplementary material, which is available to authorized users.

## Introduction

Grape must inoculation with selected *S. cerevisiae* strains is a general winemaking practice which requires the production of ADY as stable starter for long-term storage. However, this is a stressing process that challenges the fermentation performance of final biomass (Pérez-Torrado et al. 2009; Gómez-Pastor et al. 2010; Garre et al. 2010), largely due to oxidative damage of cellular macromolecules caused by ROS (reactive oxygen species) production, that influence cell vitality and viability (França et al. 2007). The adaptation and resistance of *S. cerevisiae* to desiccation include the protection against oxidative stress through ROS scavenging enzymes, such as catalase and glutathione reductase, and protective metabolites, such as trehalose and reduced glutathione (GSH). Recently, we identified a set of biochemical parameters (levels of oxidized glutathione and trehalose, and catalase and of glutathione reductase activities) after dehydration to predict physiologically relevant phenotypes for wine yeasts of potential interest for ADY production (Gamero-Sandemetrio et al. 2014), and demonstrated that low level of oxidative defense characterizes the worse performing strains. So, the analysis of protective mechanisms and/or treatments against oxidative stress, for instance via natural or food grade antioxidants, would have important biotechnological implications for the production of ADY and may also suggest potential therapeutic targets for several oxidative stress-related diseases (Halliwell and Gutteridge 2010; Escoté et al. 2012).

Several phytochemicals from fruits and vegetables may have antioxidant effects. Quercetin, the most common diet flavonol (Erlund 2004), eliminates free radicals (Van Acker and Van den Berg 1996; Ferrali et al. 1997; Belinha et al. 2007), thus preventing lipid peroxidation and protein carbonylation. Quercetin increases yeast chronological aging, which is shortened by the oxidative stress (Belinha et al. 2007). L-ascorbic acid (vitamin C), acts as reducing substrate for peroxidases (Monteiro et al. 2007). Yeasts synthesize the analogue, erythroascorbate, which prevents apoptosis induced by pro-oxidants, increases the levels of reduced glutathione and reduces ROS levels (Amari et al. 2008). Caffeic acid has antibacterial, antiviral, anti-inflammatory, anticancer and antioxidant activities (Kimura et al. 1985; Son and Lewis 2002; Wu et al. 2011; Ozturk et al. 2012). At low doses, it suppresses lipid peroxidation (Kimura et al. 1985) and blocks ROS (Wu et al. 2011). In yeast under exogenous oxidative stress, caffeic acid increases GSH levels and diminishes ROS levels (Amari et al. 2008). Supplementation of media for growth of wine yeasts with oleic acid and ergosterol can alleviate oxidative stress during must fermentation (Landolfo et al. 2010), as lipid composition of the cell membrane modulates the activity of enzymes and membrane-associated transporter functions (Avery et al. 1995; Vigh et al. 1998). Finally, resveratrol (Escoté et al. 2012) and terpene β-carotene polyphenols (Amari et al. 2008) are also of interest for their antioxidant properties in yeast.

Glycine betaine (GB) and l-proline are also found in literature as antioxidant. GB decreases ROS levels by induction of superoxide dismutase, catalase and glutathione peroxidase (Liu et al. 2011). l-proline stimulates the unfolded protein response (UPR response), thus improving the protection against oxidative damage (Liang et al. 2014).

The presence of antioxidant compounds in human diet is then widely accepted as beneficial and the use of such functional ingredients has been increased. Argan oil is an example of natural product rich in antioxidants, now commercialized both in cosmetics and food grades, which displays antiproliferative, antidiabetic, and cardiovascular risk-preventive effects (Yaghmur et al. 2001; Bennani et al. 2007).

Despite the wide use of such a variety of chemicals and natural products with antioxidant capacity for human diet and health, little biotechnological use has been described in the food fermentation industry. The aim of this study was to determine whether the antioxidant properties of pure chemicals and natural products are able of diminishing the detrimental effects of oxidative stress associated to the production of ADY for enological purposes. To do this, molasses for biomass propagation was supplemented with the aforementioned antioxidants and the obtained ADY was assayed for fermentative performance and for biochemical parameters in order to characterize also the molecular effects of the potential protectors.

## Materials and methods

### Strains and cultivation conditions

Seven natural *Saccharomyces* wine yeast strains were studied: the well-known commercial T73 wine yeast (Querol et al. 1992), the previously described *S. cerevisiae* strains (D18, D128, D170 and D301), *S. bayanus* var. uvarum (*S. u.*) (D272) and *S. cerevisiae x S. bayanus* var. uvarum (*S. c. x S. u.*) hybrid (P6) from Lallemand Inc. (Montreal, Quebec, Canada) (Gamero-Sandemetrio et al. 2013).

Precultures for the biomass propagation experiments were prepared in YPD liquid medium (1 % yeast extract, 2 % peptone, 2 %glucose) and were incubated at 30 °C with shaking (250 rpm). Liquid medium YPGF (1 % yeast extract, 2 % peptone, 10 % glucose, 10 % fructose) was used for the fermentative capacity assays. Molasses medium, diluted to 60 g/L sucrose, was supplemented with 7.5 g/L (NH_4_)_2_SO_4_, 3.5 g/L KH_2_PO_4_, 0.75 g/L MgSO_4_·H_2_O and 10 mL/L vitamin solution. Molasses and mineral solutions were autoclaved separately. The vitamin solution, containing 50 mg/L D-biotin, 1 g/L calcium pantothenate and 1 g/L thiamine hydrochloride, was filter-sterilized (0.2 μm pore size). The molasses medium was used for the biomass propagation experiments and cells were cultivated at 30 °C with shaking (250 rpm) for 24 h.

Molasses medium was supplemented with 0.20 mg/mL quercetin, 50 µg/mL resveratrol, 50 µM ascorbic acid, 50 µM β-carotene, 5 µM caffeic acid, 6 mg/mL oleic acid, 15 mg/mL ergosterol, 5 mM glycine betaine, 5 mM glutathione or 5 mM l-proline. Argan oil was added in ratio 1:100 (v/v) providing a final concentration of 6 mg/mL oleic acid in molasses.

### Biomass dehydration and rehydration conditions

Yeast biomass was separated from molasses medium by centrifugation and subjected to several washing steps with distilled water. Concentrated biomass (500 mg) was spread in open Petri dishes and dehydrated under air flux in an oven at 30 °C until approximately 8 % relative humidity (during 24 h) and kept at room temperature. For rehydration, distilled water was used to resuspend dried biomass at 37 °C for 10 min under static conditions followed by 10 min with shaking at 140×*g* (Rodríguez-Porrata et al. 2008; Garre et al. 2010; Gamero-Sandemetrio et al. 2013).

### Fermentative capacity measurement

Fresh cells and dry cells were rehydrated and inoculated (10^7^ cells/mL) in YPGF medium as described (Gamero-Sandemetrio et al. 2013). CO_2_ production was measured at 10 min intervals for 6 h in a Fermograph (ATTO Corporation, Tokyo, Japan). Fermentative capacity was expressed as mL of CO_2_ produced per 10^7^ cells. Experiments were carried out in triplicate.

### Glutathione and intracellular trehalose determination

Extracts were obtained from 100 mg of cells and used for glutathione and trehalose determination as described (Griffith 1980; Gómez-Pastor et al. 2010) and (Garre et al. 2010; Gómez-Pastor et al. 2012), respectively. The amount of glutathione was expressed as nmol per mg of cells. The amount of trehalose is given as µg of trehalose per mg of dry cell weight. Experiments were carried out in triplicate.

### Catalase and glutathione reductase activities

Extracts were obtained from 50 mg of cells and assayed spectrophotometrically as described by Jakuboswski and colleagues (Griffith 1980; Jakubowski et al. 2000) for catalase activity and as described by Murshed and colleagues (Murshed et al. 2008) for glutathione reductase activity (GR). Catalase activity was expressed as μmol of H_2_O_2_ min^−1^ mg of protein^−1^ (U/mg prot). GR activity was expressed as μmol of oxidized glutathione (GSSG) min^−1^ mg of protein^−1^ (U/mg prot).

### Measurement of protein carbonylation

Protein carbonylation in crude extracts was measured by dinitrophenilhydrazine (DNPH) derivatization and western immunodetection of protein-bound 2,4-dinitrophenylhydrazones, as previously described (Levine et al. 1994; Gómez-Pastor et al. 2010). The anti-2,4-dinitrophenol antibody (Sigma, St. Louis, Missouri, USA) was used at a 1/3500 dilution and the secondary antibody (goat anti-rabbit HRP conjugated, Amersham, Buckinghamshire, UK) was used at a 1/5000 dilution. Signals in blots were visualized using Lumigen TMA-6 (Amersham, Buckinghamshire, UK), images were captured using the Las1000 software (FujiFilm, Tokyo, Japan) and protein carbonylation was measured by image analysis using QuantityOne software (BioRad, California, USA).

### Measurement of lipid peroxidation

Quantification of lipid peroxidation was carried out by reaction of thiobarbituric acid with the malondialdehyde (MDA) product of oxidized fatty acid breakage, as previously described (Gómez-Pastor et al. 2010). Lipid peroxidation was expressed as pmoles of MDA mg of cells^−1^.

### Statistical analysis

Sample averages were compared using a Student’s *t* test. The samples denoted (a) were significantly different from those labelled (b) with a *p* < 0.05, and also different from those denoted (c) with a *p* < 0.05. The samples labelled (ab) were not significantly different from (a) and (b), but were significantly different from (c). The samples denoted (*) were significantly different from each other.

A multivariate analysis (general linear model) assessed the effect of the supplementation with different antioxidant and strain on oxidative stress parameters (biomass propagation, fermentation capacity, lipid peroxidation, protein carbonylation, protective metabolites and enzymatic activities). The results were statistically compared by using 2-way ANOVA and the Tukey HSD post hoc test. Statistical hypothesis tests were used to check the null hypotheses (α = 0.05) (SPSS v22.0; IBM SPSS Inc). Moreover, PCA (Principal Component Analysis) was generate to visualize a 2D plot of the first two principal components, revealing potential grouping patterns among supplementations or facilitating the recognition of outlier groups using PAST 3.05 statistical software package (Hammer et al. 2001).

## Results

### Ascorbic, caffeic and oleic acids supplementation increases biomass yield, reduces oxidative damage, and improves fermentative capacity

A set of previous experiments were conducted in order to select the best antioxidants and their optimal concentrations (data not shown). Only quercetin, reduced glutathione (GSH), and caffeic, ascorbic, oleic acids improved the biomass yield (Table 1 and Additional file 1: Table S1), being the three acids widely effective. Cold-adapted strains D128, D272 and P6 (Salvadó et al. 2011) were also benefited by quercetin, and two of them by GSH (Additional file 1: Table S1). However, the effect of the antioxidants on fermentative capacity was more complex in terms of strain dependency (Table 1). Only D18 and D128 strains were not improved by any treatment and a variety of positive effects was obtained: strains D272 (*S. u*) and P6 (*S. c x S. u*) displayed higher fermentative capacity when grown in molasses supplemented with ascorbic, caffeic or oleic acids, while strain D301 with ascorbic (2.31 ± 0.88 fold) or oleic acid (2.18 ± 0.12 fold); strain D170 with caffeic (1.06 ± 0.06 fold) and oleic acids (1.15 ± 0.01 fold) and strain T73 with ascorbic acid (2.11 ± 0.12 fold) and quercetin (1.42 ± 0.33 fold). Again, all three acids were the best performers, with 4 out of 7 strains improving their fermentative capacity in the presence of ascorbic or oleic acids. Quercetin proved to produce the worst performance, reducing in fact the fermentative capacity of two strains and only improving it in the case of T73 strain (Additional file 1: Table S1).

**Table 1:** Technological properties and oxidation biomarkers of ADY from *Saccharomyces* wine strains under molasses supplementation with different antioxidants

Parameter	Control	5 µM Caffeic acid	6 mg/mL Oleic acid	5 µM Ascorbic acid
T73				
Biomass (OD_600_)	18.42 (±0.2)	*28.43 (±1.1)*	*33.36 (±2.1)*	*27.03 (±0.7)*
FC (mL CO_2_/10^7^cells)	10.52 (±0.3)	9.1 (±0.7)	9.03 (±1.7)	22.21 (±2.4)
LP (pmol MDA/mg protein)	26.85 (±1.0)	*21.32 (±1.6)*	29.02 (±0.8)	23.50 (±1.0)
GSH (nmol/mg cell)	1.67 (±0.01)	*1.97 (±0.12)*	*2.45 (±0.02)*	*2.23 (±0.01)*
Trehalose (µg/mg cells)	100.1 (±9.7)	*547.2 (±2.9)*	*649.7 (±3.8)*	*940.6 (±3.1)*
Δ GR (U/mg prot)	−0.52 (±0.3)	*0.88 (±0.15)*	*1.08 (±0.41)*	*0.35 (±0.11)*
Δ Catalase (U/mg prot)	18.96 (±0.1)	*9.86 (±2.1)*	*15.42 (±1.4)*	*5.49 (±0.7)*
D18				
Biomass (OD_600_)	16.64 (±0.1)	*37.41 (±0.1)*	*26.58 (±0.7)*	*28.81(±1.1)*
FC (mL CO_2_/10^7^cells)	8.39 (±0.9)	9.55 (±0.9)	9.78 (±0.8)	6.75 (±0.6)
LP (pmol MDA/mg protein)	24.11 (±2.3)	*9.40 (±1.8)*	*19.87 (±0.9)*	*17.09 (±1.3)*
GSH (nmol/mg cell)	0.91 (±0.02)	*1.27 (±0.01)*	*2.65 (±0.03)*	*1.50 (±0.02)*
Trehalose (µg/mg cells)	193.5 (±10)	*765.2 (±3.9)*	*287.5 (±9.4)*	*853.6 (±3.3)*
Δ GR (U/mg prot)	−0.17 (±0.1)	*1.07 (±0.18)*	*1.34 (±0.32)*	*0.25 (±0.16)*
Δ Catalase (U/mg prot)	31.26 (±0.7)	*9.77 (±2.2)*	*4.87 (±0.9)*	*11.22 (±1.9)*
D170				
Biomass (OD_600_)	17.61 (±0.2)	*28.26 (±0.1)*	*26.40 (±1.1)*	*21.95 (±0.6)*
FC (mL CO_2_/10^7^cells)	17.18 (±0.1)	*18.31 (±1.5)*	*19.86 (±0.7)*	*8.52 (±0.4)*
LP (pmol MDA/mg protein)	23.34 (±0.9)	*7.69 (±1.2)*	*21.15 (±0.5)*	*46.58 (±0.9)*
GSH (nmol/mg cell)	1.24 (±0.03)	*1.22 (±0.07)*	*1.23 (±0.10)*	*1.19 (±0.08)*
Trehalose (µg/mg cells)	155.5 (±2.8)	*204.6 (±5.9)*	*376.7 (±4.9)*	*442.6 (±1.0)*
Δ GR (U/mg prot)	2.57 (±0.7)	3.96 (±0.59)	*4.90 (±0.02)*	*2.33 (±0.21)*
Δ Catalase (U/mg prot)	0.28 (±1.7)	11.25 (±3.8)	8.89 (±0.04)	7.26 (±2.4)
D301				
Biomass (OD_600_)	17.66 (±0.4)	*22.68 (±0.1)*	*27.53 (±1.1)*	*22.05 (±0.1)*
FC (mL CO_2_/10^7^cells)	3.58 (±0.1)	1.3 (±0.1)	*7.83 (±0.5)*	*8.26 (±1.1)*
LP (pmol MDA/mg protein)	20.08 (±1.7)	19.79 (±0.9)	20.08 (±0.2)	19.48 (±0.8)
GSH (nmol/mg cell)	1.20 (±0.05)	1.14 (±0.06)	1.10 (±0.12)	1.19 (±0.06)
Trehalose (µg/mg cells)	141.1 (±22)	14.5 (±21)	38.5 (±3.5)	37.9 (±1.4)
Δ GR (U/mg prot)	0.89 (±0.23)	1.51 (±0.23)	1.28 (±0.23)	0.88 (±0.01)
Δ Catalase (U/mg prot)	67.76 (±0.4)	*6.00 (±0.4)*	*5.57 (±0.4)*	*7.26 (±0.4)*
D128				
Biomass (OD_600_)	14.11 (±0.1)	*29.48 (±0.1)*	*26.95 (±2.1)*	*16.74 (±0.2)*
FC (mL CO_2_/10^7^cells)	2.33 (±0.6)	1.75 (±0.1)	1.68 (±0.1)	1.88 (±0.2)
LP (pmol MDA/mg protein)	22.00 (±1.8)	*10.04 (±1.4)*	*19.02 (±0.5)*	*22.22 (±0.8)*
GSH (nmol/mg cell)	0.43 (±0.05)	*1.26 (±0.13)*	*1.27 (±0.08)*	*1.35 (±0.07)*
Trehalose (µg/mg cells)	153.8 (±11)	144.9 (±7.6)	*202.4 (±22)*	*309.4 (±3.8)*
Δ GR (U/mg prot)	1.70 (±0.28)	*2.30 (±0.09)*	*4.60 (±0.29)*	*3.10 (±0.01)*
Δ Catalase (U/mg prot)	0.39 (±1.1)	6.71 (±1.13)	4.91 (±1.0)	7.56 (±0.13)
D272				
Biomass (OD_600_)	11.26 (±0.2)	*21.02 (±0.2)*	*27.81 (±1.9)*	*8.59 (±1.2)*
FC (mL CO_2_/10^7^cells)	10.42 (±0.4)	*23.51 (±1.4)*	*13.15 (±0.6)*	*20.29 (±2.2)*
LP (pmol MDA/mg protein)	20.83 (±1.4)	*10.04 (±1.0)*	*6.41 (±0.1)*	*14.96 (±0.4)*
GSH (nmol/mg cell)	2.42 (±0.22)	*1.38 (±0.15)*	1.26 (±0.08)	1.30 (±0.06)
Trehalose (µg/mg cells)	190.9 (±2.5)	*361.4 (±10)*	*394.4 (±17)*	*724.8 (±10)*
Δ GR (U/mg prot)	1.10 (±0.20)	*4.70 (±0.31)*	*1.80 (±0.39)*	*2.80 (±0.02)*
Δ Catalase (U/mg prot)	−2.24 (±0.3)	1.70 (±1.26)	1.59 (±0.24)	1.52 (±0.30)
P6				
Biomass (OD_600_)	13.72 (±1.1)	*30.94 (±0.1)*	*48.62 (±0.1)*	*23.81 (±0.2)*
FC (mL CO_2_/10^7^cells)	12.53 (±0.6)	*14.52 (±0.8)*	*17.65 (±0.1)*	*16.37 (±0.7)*
LP (pmol MDA/mg protein)	22.50 (±0.9)	*13.46 (±1.5)*	*15.38 (±1.6)*	*15.38 (±1.9)*
GSH (nmol/mg cell)	3.65 (±0.38)	1.43 (±0.02)	2.14 (±0.13)	1.74 (±0.28)
Trehalose (µg/mg cells)	252.6 (±24)	235.7 (±1.4)	*691.3 (±40)*	*828.8 (±4.3)*
Δ GR (U/mg prot)	1.20 (±0.01)	2.40 (±0.39)	3.7 (±0.76)	3.10 (±0.27)
Δ Catalase (U/mg prot)	0.064 (±0.1)	0.80 (±0.10)	0.14 (±0.20)	0.40 (±0.10)

Italics indicates significant difference respect to the control from non-supplemented molasses with *p* < 0.05. In brackets SD value from three independent experiments

*FC* fermentative capacity, *LP* lipid peroxidation, *GSH* reduced glutathione, *GR* glutathione reductase activity

Lipid peroxidation was measured as oxidative damage marker. In general, ascorbic, caffeic and oleic acids diminished lipid peroxidation after dehydration (Table 1), being caffeic acid the best protector except for strains D272 (*S. u*), which responded better to oleic acid, and D301, which lipids were not protected by any antioxidant, being in fact more damaged by two of them, quercetin and GSH.

**Fig. 1:**
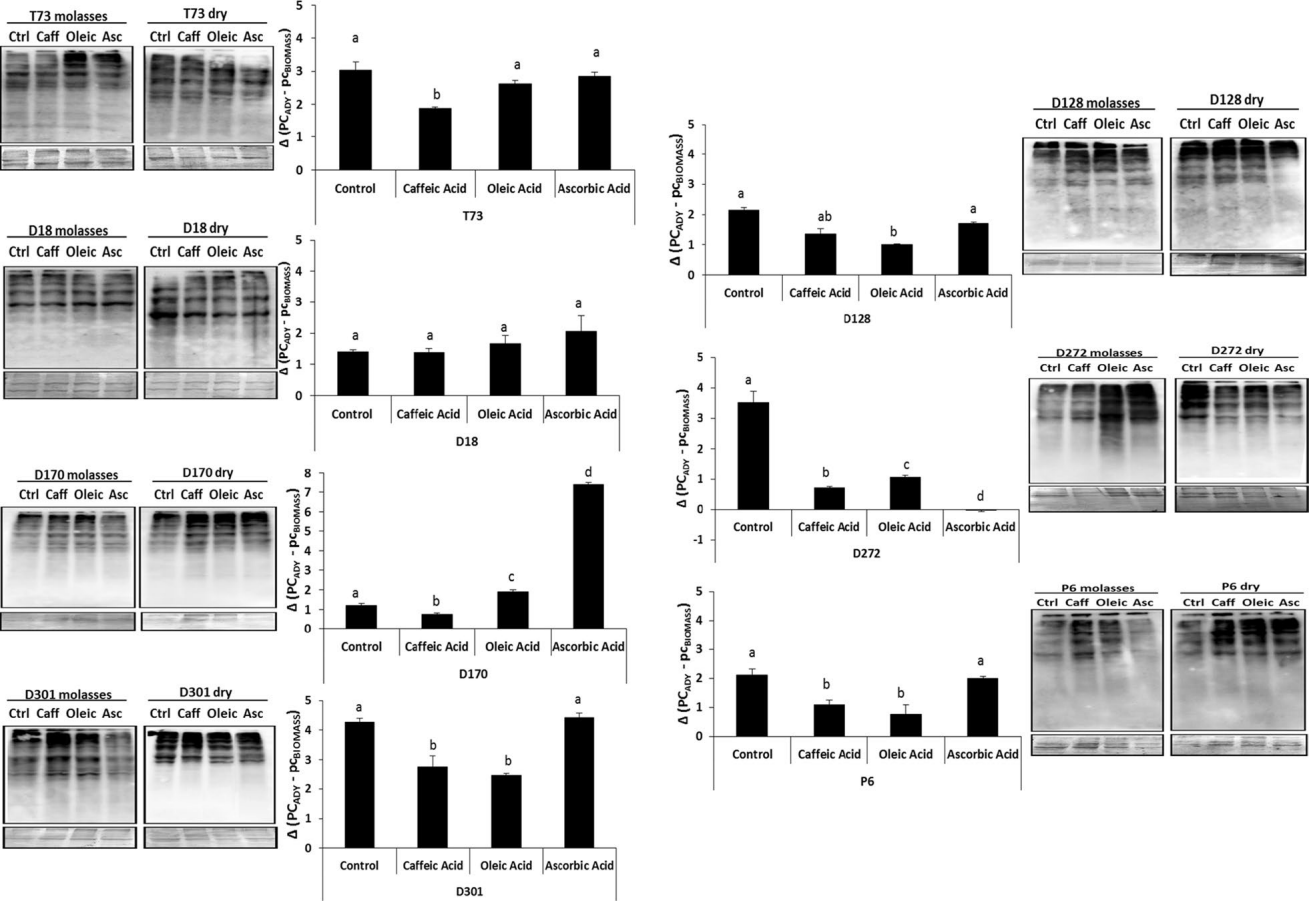
Protein carbonylation after dehydration. PC_ADY_ (protein carbonylation in dry cells); PC_BIOMASS_ (protein carbonylation in fresh cells). Protein carbonylation westerns and quantification as Ci/Pi, where Ci is protein carbonyl content quantified by image analysis and Pi is total protein from the coomassie-stained membranes. Error bars correspond to the SD of three independent experiments. (*a*) was significantly different from (*b*, *c*, *d*) with a *p* < 0.05. The sample labeled as (*ab*) was not significantly different from (*a*) and (*b*), but was significantly different from (*c*, *d*)

Protein carbonylation, other marker of oxidative insult, was then measured in fresh and dried biomass from molasses supplemented with ascorbic, caffeic and oleic acid, the best antioxidants in terms of lipid protection (Fig. 1). In general, caffeic acid and oleic acids diminished the degree of protein carbonylation after dehydration in a strain-dependent manner, while ascorbic acid may even increase it, as seen for strain D170 (6.09 ± 0.12 fold), which is consistent with the reduction in fermentative capacity and the increase in lipid peroxidation after ascorbic acid supplementation.

### Improved post-dehydration performance by antioxidants supplementation is predictable from selected biomarkers

Previously described predictive parameters for ADY performance (Van Acker and Van den Berg 1996) were measured after antioxidant supplementation. Intracellular reduced glutathione (GSH) levels, indicative of the redox state (Grant et al. 1996; Espindola et al. 2003) are shown in Table 1. All the antioxidants increased GSH level in T73 and D18 strains, decreased it in D272 and P6 strains and did not affect D170 and D301 strains. According to data from both total glutathione and GSSG (data not shown), the antioxidants be acting at the level of synthesis of glutathione. The effect of GSH supplementation on intracellular GSH level may be biased and, then, it is not considered.

Accumulation of intracellular trehalose, indicative of stress response (Pereira et al. 2003), was also measured (Table 1). In general, ascorbic and oleic acids increased trehalose levels in ADY, except for D301 strain, which reduced trehalose content under any antioxidants supplementation. In opposite way, T73 and D272 strains increase their trehalose contents under any antioxidant addition.

Regarding antioxidant enzymes, Table 1 shows the increment of glutathione reductase (GR) activity and catalase activity between fresh and dry cells. In general, ascorbic, caffeic and oleic acids induced GR activity, except for D170 and, again, D301 strains, in agreement with the observed GSH levels. For catalase activity, D170 and D128 strains displayed the highest induction with all the antioxidants (Table 1, Addtional file 1: Table S1). The diminished activity in T73, D18 and D301 seems to be related to a higher increment in control conditions, and strains D272 and P6 displayed very low basal levels of activity but significant induction after treatments.

Finally, a statistical principal component analysis (PCA) was performed for each strain in order to generate 2-dimensional graphics (2D-plot) that reveals potential grouping patterns between supplementations and facilitates recognition performed atypical groups using the statistical software package PAST 3.05 (Additional file 1: Fig. S1). In general, although ascorbic, caffeic and oleic acid molecules improved the physiological performance of the obtained LSA, our results indicate that the antioxidant capacity of these compounds is strain dependent and the molecular protection in the biomass does not necessarily leads to increased fermentative capacity.

### Argan oil supplementation mimics the beneficial effects and also the biomarker patterns of antioxidants in ADY production

Argan oil was then selected as a natural compound due to its high content in ascorbic, caffeic and oleic acids, and T73 and P6 strains (with high and low biomass yield in control situation, respectively) were used to find the adequate concentration to reverse the growth defects caused by 4 mM H_2_O_2_ addition (data not shown) in molasses supplemented with 4, 8, 6, 20 and 40 mg/mL of argan oil. 6 mg/mL was finally selected, as described in Materials and Methods.

**Fig. 2:**
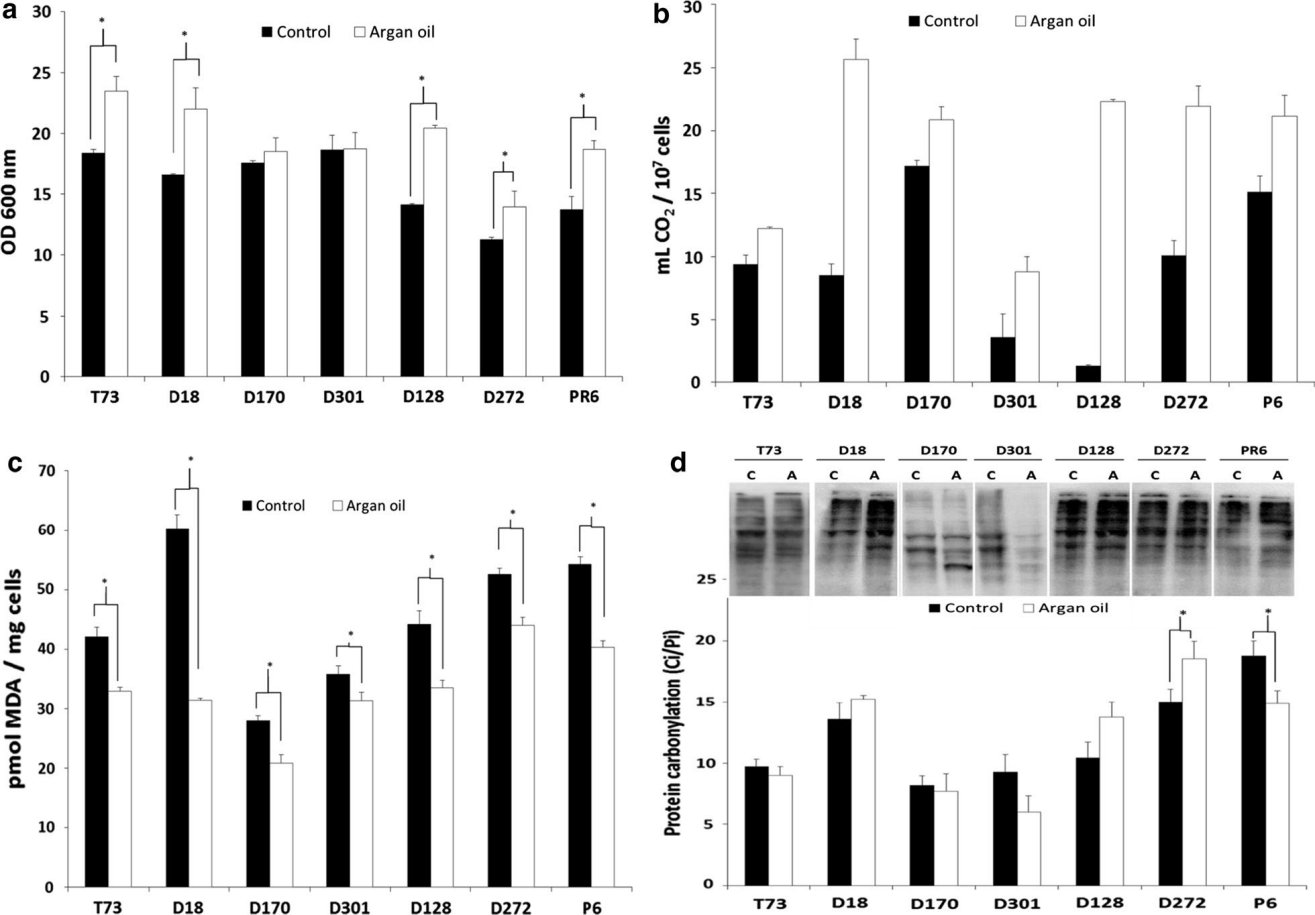
Effects of argan oil supplementation in physiological performance and oxidative damage. **a** Biomass yield. **b** Fermentative capacity. **c** Lipid peroxidation in dry cells was expressed as the amount of MDA per mg of protein. **d** Protein carbonyl content as in Fig. 1. *Error bars* correspond to the SD value of three independent experiments. *Asterisk* are significantly different from control (non-supplemented molasses) with *p* < 0.05

Supplementation of molasses with 6 mg/mL of argan oil increased biomass yield and fermentative capacity (Fig. 2a, b, respectively), except for D301 and D170 strains, which were improved only in the second parameter. Lipid peroxidation was diminished by argan oil in agreement to its beneficial effects (Fig. 2c). Protein carbonylation (Fig. 2d), however, was in general not affected by argan oil, except for D272 and P6, were contrary effects are observed without a correlation with biomass yield or fermentative capacity.

**Fig. 3:**
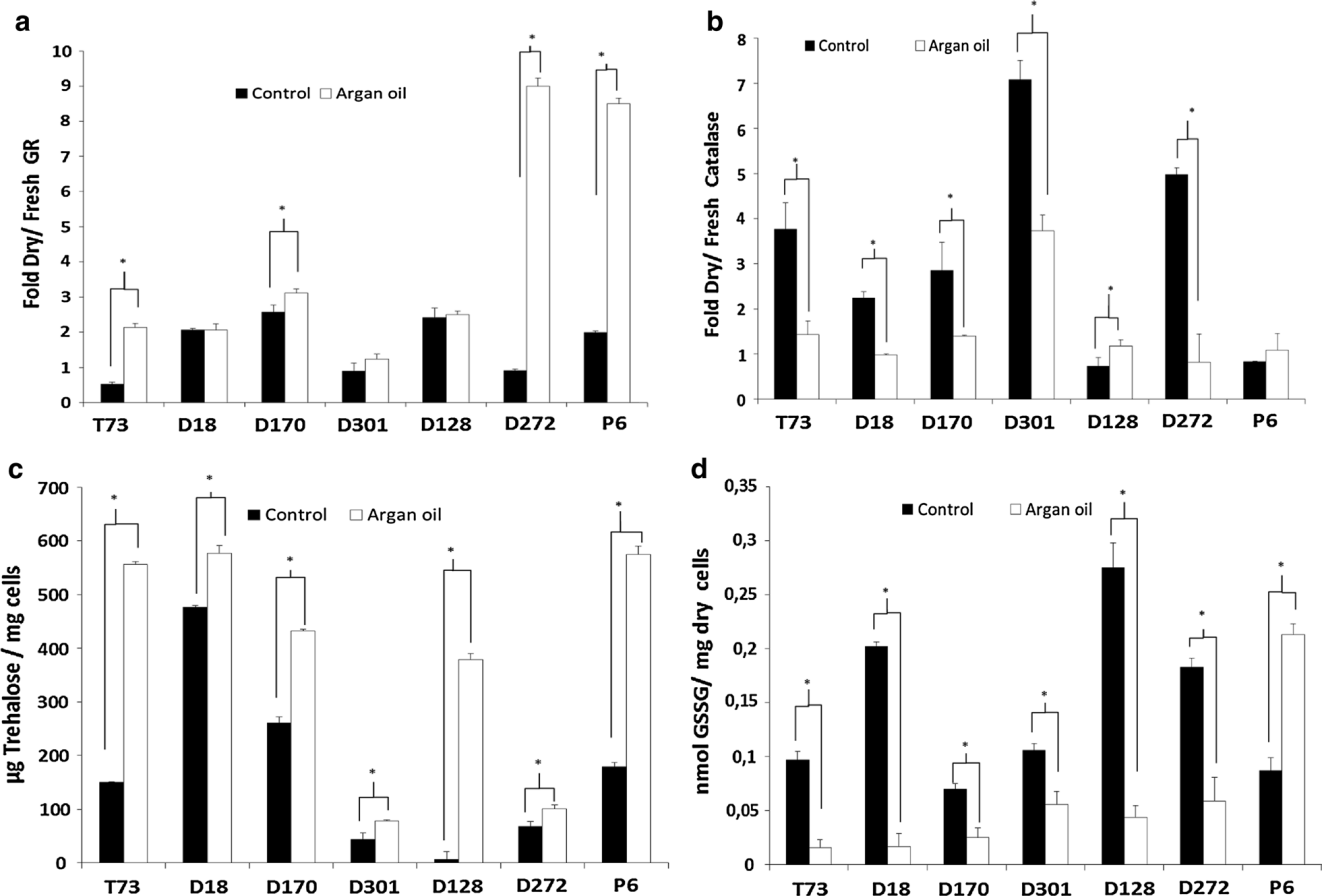
Analysis of predictive biomarkers in ADY after argan oil supplementation during biomass propagation. **a** Glutathione reductase (GR) and **b** catalase activities. **c** Trehalose content. **d** Oxidized glutathione. *Errors bars* correspond to the SD of three independent experiments. *Asterisk* was significantly different from the control with a *p* < 0.05

The aforementioned predictive biomarkers were also analyzed for ADY obtained under argan oil supplementation (Fig. 3). Overall, higher induction of glutathione reductase activity (Fig. 3a) and lower induction of catalase activity (Fig. 3b) were observed, similarly to what was observed with pure chemicals. Additionally, trehalose levels (Fig. 3c) were increased in all the strains and GSSG levels (Fig. 3d) decreased, except for P6 strains which displayed overaccumulation of oxidized, but also of reduced glutathione (data not showed). According to data from both total glutathione and GSH (data not shown), argan oil would stimulate the glutathione synthesis.

**Fig. 4:**
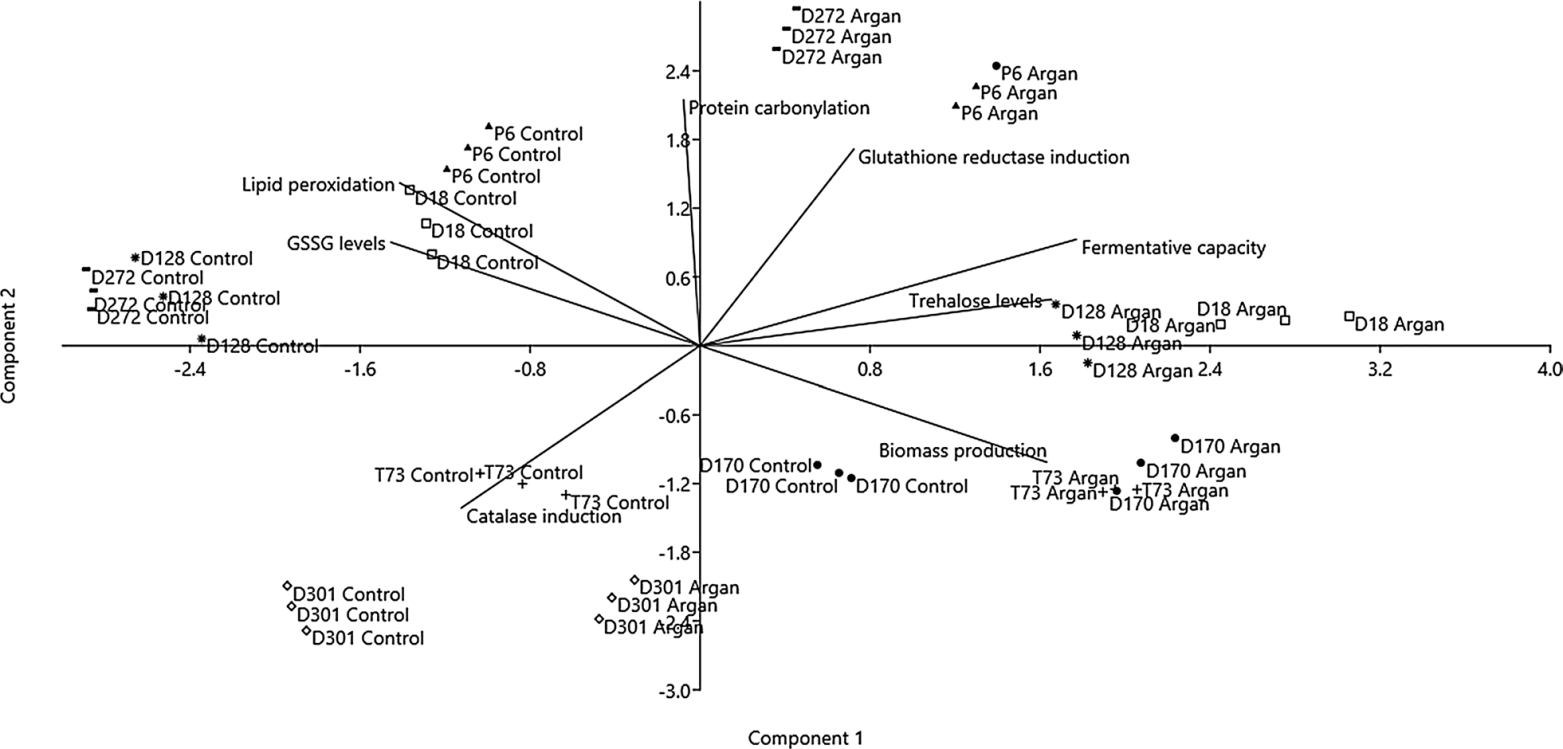
Principal components (PCA) statistical analysis of the argan oil effects on the physiological and biochemical biomarkers with a total variance represented of 70 %. Component 1 reflects a 39.55 % total variance (with a positive correlation with biomass yield, fermentative capacity and trehalose levels) and Component 2 reflects a 30.45 % total variance (with a positive correlation with glutathione reductase activity). The lines belong to variance of dependent variables or biochemical biomarkers measured (biomass yield, fermentative capacity, lipid peroxidation, protein carbonylation, protective metabolites and enzymatic activities) arranged in two dimensions according to the components 1 and 2. Study strains and conditions (control and argan oil supplementation) are labelled with different symbols: T73 (**+**); D18 (*unfilled square*); D170 (*filled circle*); D301 (*unfilled diamond*); D128 (*Asterisk*); D272 (−); P6 (*triangle*) and they appear associated with the dependent variable that differs from other strains and conditions

Finally, a statistical principal component analysis (PCA) was performed and the 2-dimensional graph (2D-plot) was used to better define the effect of supplementation with argan oil on physiological and oxidative stress parameters (Fig. 4). As can be seen, samples from the treatment with argan oil, regardless of the strain (except for D301), appear in the right of the graphic and directly correlate to further increases, compared the control situation, of the parameters: induction of glutathione reductase activity, fermentation capacity, trehalose levels and biomass production. Furthermore, there is an inverse correlation with the parameters: induction of catalase activity, GSSG levels and lipid peroxidation. More specifically, we can identify the main effect exerted for supplementation with argan oil on each strains, where: T73 and D170 would increase further its biomass production; D128 and D18 trehalose levels; D272 protein carbonylation and P6 glutathione reductase activity; these results correlate with the data observed in the above experiments (Figs. 2, 3).

Overall, these data correlate with the improvement in biomass yield and fermentative performance of ADY after argan oil supplementation.

## Discussion

The industrial process of wine yeast biomass dehydration affects the viability and vitality of yeast cells (Matthews and Webb 1991), which is largely due to oxidative damage of cellular components caused by ROS production (França et al. 2007). We have recently described that wine strains with naturally high antioxidant defenses, suffer low oxidative damage and display high fermentative capacity after dehydration, and we have tested easily predictable markers for this biotechnologically relevant behavior (Gamero-Sandemetrio et al. 2014).

In this study, the known antioxidant properties of a collection of pure chemicals have been exploited to improve the efficiency of ADY production by molasses supplementation. Seven oenological interesting strains were analyzed after biomass propagation and dehydration, and the yield was determined and the ADY product was assayed for fermentative capacity, macromolecular damage and the four predictive biomarkers (Gamero-Sandemetrio et al. 2014).

In general, single ascorbic, caffeic or oleic acids supplementation were found to be the most effective as it increases biomass yield and fermentative performance, diminishes oxidative macromolecular damage, increases GSH and trehalose levels, induces glutathione reductase activity and reduces catalase activity, all together predicting a greater resistance of ADY to oxidative stress. Ascorbic acid has been shown to efficiently scavenge ROS, protect membrane lipids against peroxidation (Sies and Sathl 1995) and increase reduced glutathione (GSH) levels (Amari et al. 2008). Accordingly, our results show that ascorbic acid lowers the level of lipid peroxidation, likely due to increased GSH and trehalose contents, and also to regulatory effects on the 1-Cys peroxiredoxin activity (Monteiro et al. 2007), but it does not protect protein against carbonylation.

Caffeic acid has been linked with apoptosis and anticancer and antifungal activities (Amari et al. 2008), and at low doses, it diminishes lipid peroxidation and blocks ROS. Oleic acid supplementation has been used in wine yeast to mitigate oxidative stress during must fermentation since the lipid composition of cell membranes affects the activities of membrane-associated enzymes and transporters (Landolfo et al. 2010). In this study, both antioxidants lowered lipid peroxidation and protein carbonylation after desiccation, thus improving the oxidative response of ADY. Our results also indicate that the antioxidant capacity of these compounds is strain-dependent, and that improved biomass yield does not necessarily involve improvement in fermentative capacity, as observed for D18 and D128 strains. That dependency may be useful to understand the molecular mechanisms different between strains. For instance, fermentative capacity of strains containing *S. uvarum* genome (D272 and P6) is increase by antioxidants, but the amount of reduced glutathione is reduced in a particular way by antioxidants in these strains, although GR is greatly induced by antioxidants. Therefore GSH metabolisms seems to be distinct feature of this *Saccharomyces* species, and it may be a target to improve its drying tolerance, that tends to be lower that *S. cerevisiae* (Rodríguez-Porrata et al. 2011).

Another important withdraw from this study is the complexity of cellular mechanisms in interplay under oxidative stress and the variety of specific effects of antioxidant compounds. Both factors generate a diversity of strain- and antioxidant-dependent behaviors. Physiological improvement of ADY not always correlates to changes in all the selected oxidative stress biomarkers, as is the case of D301 strain in which ascorbic and oleic acids improved its fermentative capacity but GSH level and GR activity were not modified even trehalose level had diminished. It is also worth to note that some antioxidants have not a general beneficial effect but they improve specific properties in some interesting strains, as quercetin, which improved biomass production in D272 (*S. b.*), P6 (*S. c* x *S. u*) and D128 (cold-adapted *S. c.*). As other phenolic compounds that reduce oxidative damage, it lowers lipid peroxidation and protects yeast cells from oxidative stress, likely acting on membranes through cellular signaling pathways independent of glutathione and catalase activity (Belinha et al. 2007). Those effects may be useful in the industries where the goal is produce yeast biomass as food additive.

The use of pure chemicals as protectors is expensive and may be controversial in food industries, so we propose food-grade argan oil supplementation during the propagation of biomass for wine ADY production, which reproduces their beneficial effects on fermentative capacity. Argan oil contains high levels of linoleic and oleic acids and is rich in polyphenols and tocopherols, which exhibit significant antioxidant activity (Yaghmur et al. 2001). Minor compounds, such sterols, carotenoids, caffeic acid, ascorbic acid, and squalene contribute to its nutritional, dietetic and organoleptic value, and to its preservative and health properties (Bennani et al. 2007). Argan oil improved fermentative capacity in all the strains and, in most of them, also biomass yield. The beneficial effect of argan oil can be mediated by prevention of membrane damage, as it significantly reduced lipid peroxidation, an effect that can be attributed to some components (vegetable oils, tocopherols, ascorbic acid) acting as neutralizers of lipid peroxyl radicals (Sies and Stahl 1995). Additionally, argan oil also reduced GSSG levels, raised trehalose levels, and modulated the activity of enzymatic activities such as GR and catalase.

## Additional file

10.1186/s13568-015-0159-7 Principal Components (PCA) statistical analysis of the antioxidant molecules effects (Figure S1) and Technological properties and oxidation biomarkers of ADY from *Saccharomyces* wine strains under molasses supplementation with additional antioxidants (Table S1).
