# Supplementary material for: Food-grade argan oil supplementation in molasses enhances fermentative performance and antioxidant defenses of active dry wine yeast
Source: AMB Express. 2015 Dec 1;5:75. doi: 10.1186/s13568-015-0159-7 (PMC4666183; doi:10.1186/s13568-015-0159-7)
Supplement: Supplementary file 1 — 10.1186/s13568-015-0159-7 Principal Components (PCA) statistical analysis of the antioxidant molecules effects (Figure S1) and Technological properties and oxidation biomarkers of ADY from Saccharomyces wine strains under molasses supplementation with additional antioxidants (Table S1). [file 13568_2015_159_MOESM1_ESM.pdf]

Applied Microbiology and Biotechnology

Food-grade argan oil supplementation in molasses enhances fermentative performance and antioxidant defenses of active dry wine yeast.

Esther Gamero-Sandemetrio[2], Max Torrellas[1], María Teresa Rábena[3], Rocío Gómez-Pastor[1,2], Agustín Aranda[2] and Emilia Matallana[#1,2]

[1]Departament de Bioquímica i Biologia Molecular, Universitat de València, València.

[2]Departamento de Biotecnología, Instituto de Agroquímica y Tecnología de Alimentos, CSIC, Valencia, Spain.

[3]Departament d'Estadística i Investigació Operativa. Universitat de València, València.

[#]Corresponding author: Dr. Emilia Matallana

Calle Agustín Escardino, 7. 46980 Paterna (Valencia). (+34) 963 90 0022. FAX:(+34) 963 63 6301

emilia.matallana@uv.es

A) T73

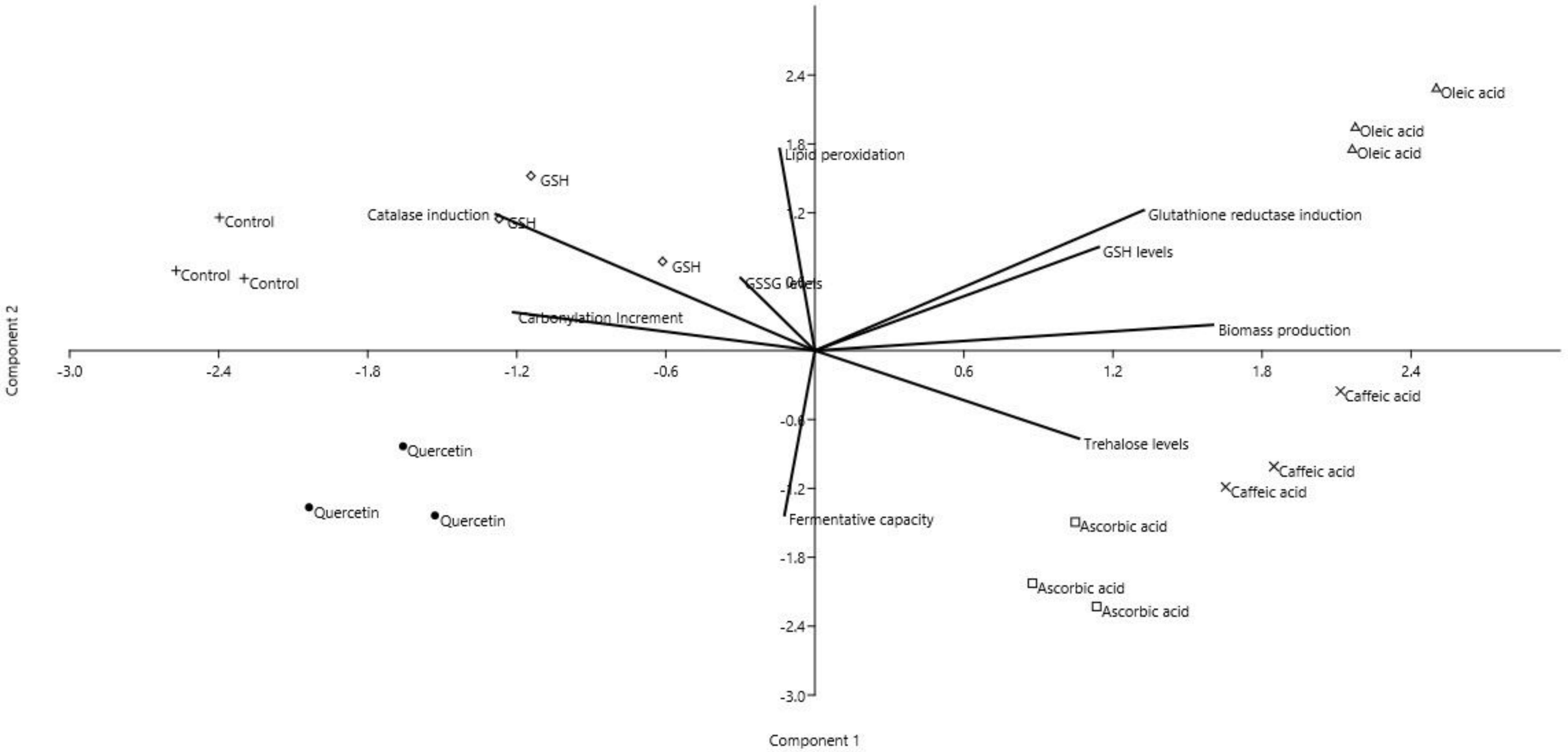

Component 2
Component 1
2.4
1.8
1.2
0.6
-0.6
-1.2
-1.8
-2.4
-3.0
0.6
1.2
1.8
2.4
Oleic acid
Oleic acid
Oleic acid
Lipid peroxidation
GSH
Catalase induction
GSH
Control
Glutathione reductase induction
GSH levels
GSH
Control
Control
GSSG levels
Carbonylation Increment
Biomass production
Caffeic acid
Quercetin
Trehalose levels
Caffeic acid
Caffeic acid
Quercetin
Quercetin
Fermentative capacity
Ascorbic acid
Ascorbic acid
Ascorbic acid

B) D18

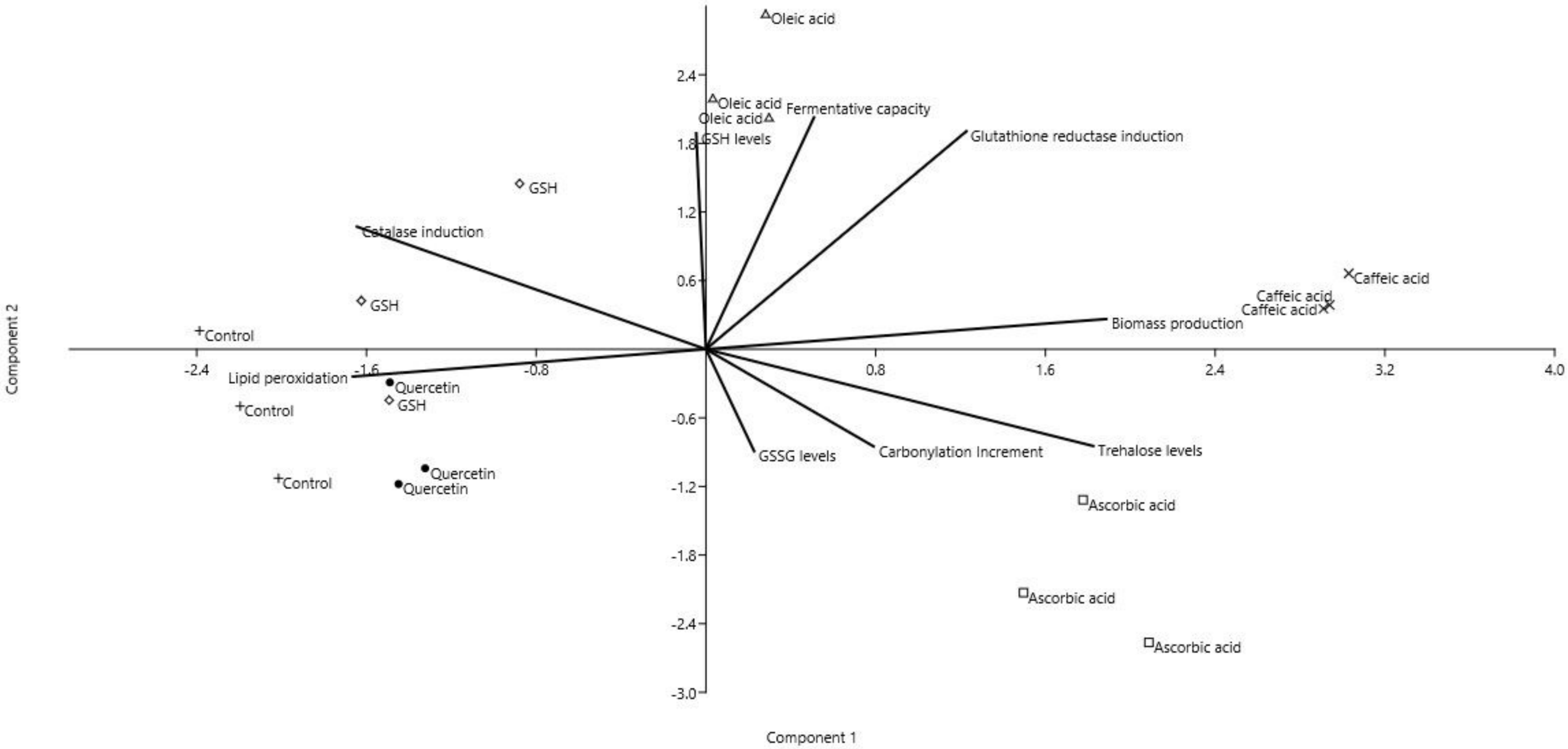
Oleic acid
Oleic acid
Oleic acid
Fermentative capacity
GSH levels
Glutathione reductase induction
GSH
Catalase induction
Caffeic acid
Caffeic acid
Caffeic acid
GSH
Control
Biomass production
Lipid peroxidation
Quercetin
GSH
Control
GSSG levels
Carbonylation Increment
Trehalose levels
Quercetin
Quercetin
Control
Ascorbic acid
Ascorbic acid
Ascorbic acid
Component 2
Component 1
2.4
1.8
1.2
0.6
-0.6
-1.2
-1.8
-2.4
-3.0
-2.4
-1.6
-0.8
0.8
1.6
2.4
3.2
4.0

C) D170

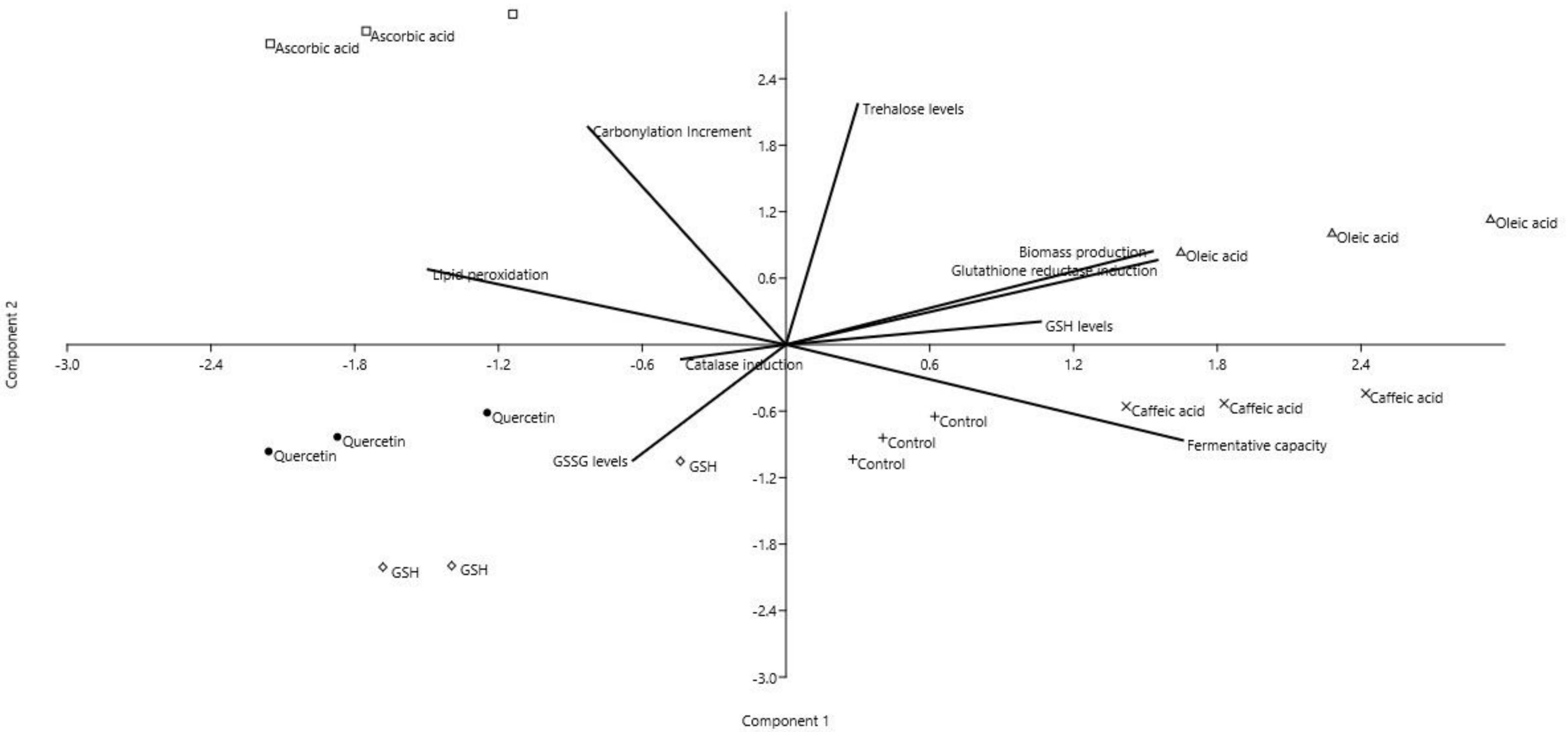

Ascorbic acid
Ascorbic acid
Trehalose levels
Carbonylation Increment
Oleic acid
Oleic acid
Oleic acid
Biomass production
Glutathione reductase induction
Lipid peroxidation
GSH levels
Catalase induction
Caffeic acid
Caffeic acid
Caffeic acid
Quercetin
Quercetin
Quercetin
Control
Control
Control
Fermentative capacity
GSSG levels
GSH
GSH
GSH
Component 1
Component 2
-3.0
-2.4
-1.8
-1.2
-0.6
0.6
1.2
1.8
2.4
-3.0
-2.4
-1.8
-1.2
-0.6
0.6
1.2
1.8
2.4

D) D301

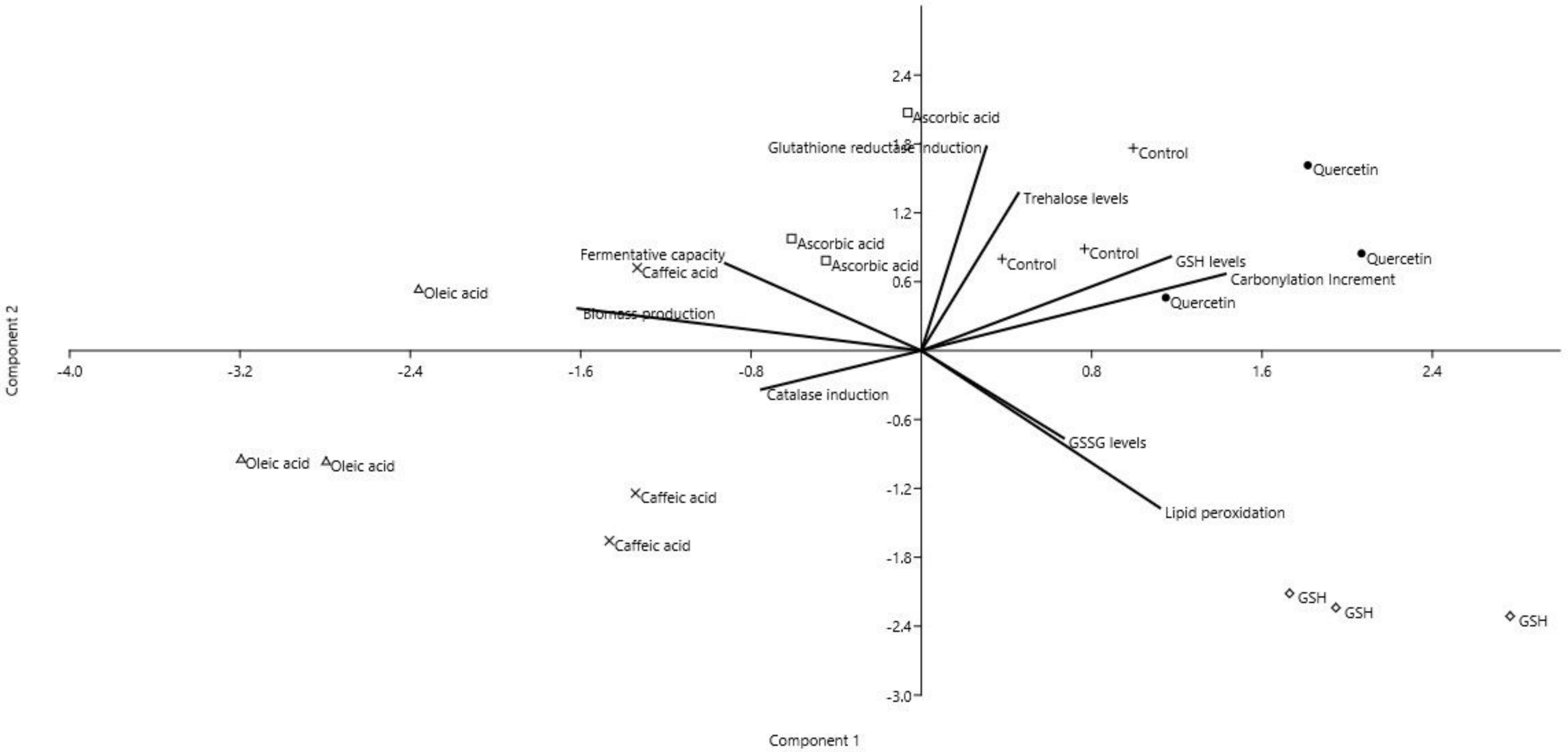

Ascorbic acid
Glutathione reductase induction
Control
Quercetin
Trehalose levels
Ascorbic acid
Fermentative capacity
Caffeic acid
Ascorbic acid
Control
Control
GSH levels
Quercetin
Carbonylation Increment
Oleic acid
Quercetin
Biomass production
Catalase induction
GSSG levels
Oleic acid
Oleic acid
Caffeic acid
Lipid peroxidation
Caffeic acid
GSH
GSH
GSH
2.4
1.8
1.2
0.6
-0.6
-1.2
-1.8
-2.4
-3.0
-4.0
-3.2
-2.4
-1.6
-0.8
0.8
1.6
2.4
Component 2
Component 1

E) D128

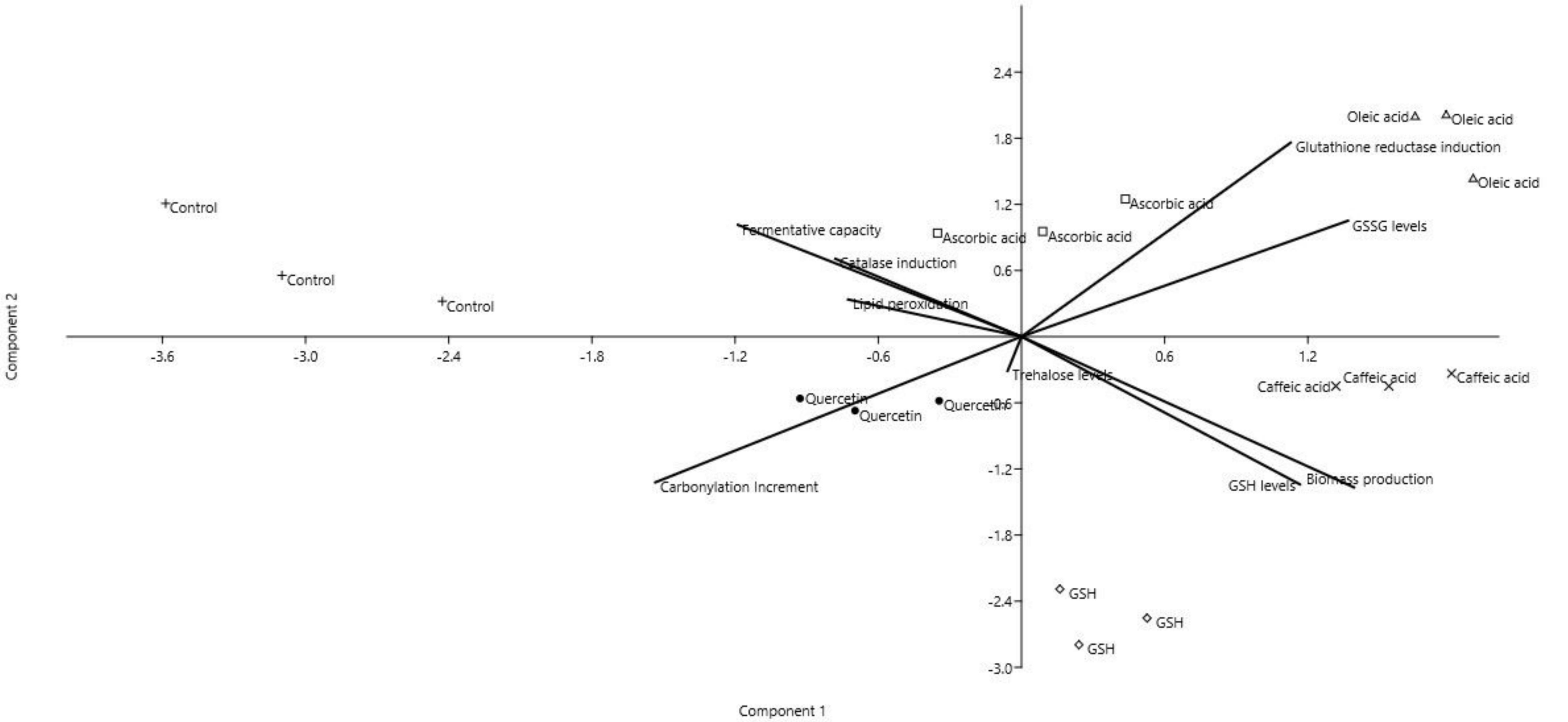

Component 2
Component 1
Control
Control
Control
Oleic acid
Oleic acid
Oleic acid
Glutathione reductase induction
Ascorbic acid
Ascorbic acid
Ascorbic acid
GSSG levels
Fermentative capacity
Catalase induction
Lipid peroxidation
Trehalose levels
Caffeic acid
Caffeic acid
Caffeic acid
Quercetin
Quercetin
Quercetin
Carbonylation Increment
GSH levels
Biomass production
GSH
GSH
GSH
-3.6
-3.0
-2.4
-1.8
-1.2
-0.6
0.6
1.2
2.4
1.8
1.2
0.6
-0.6
-1.2
-1.8
-2.4
-3.0

F) D272

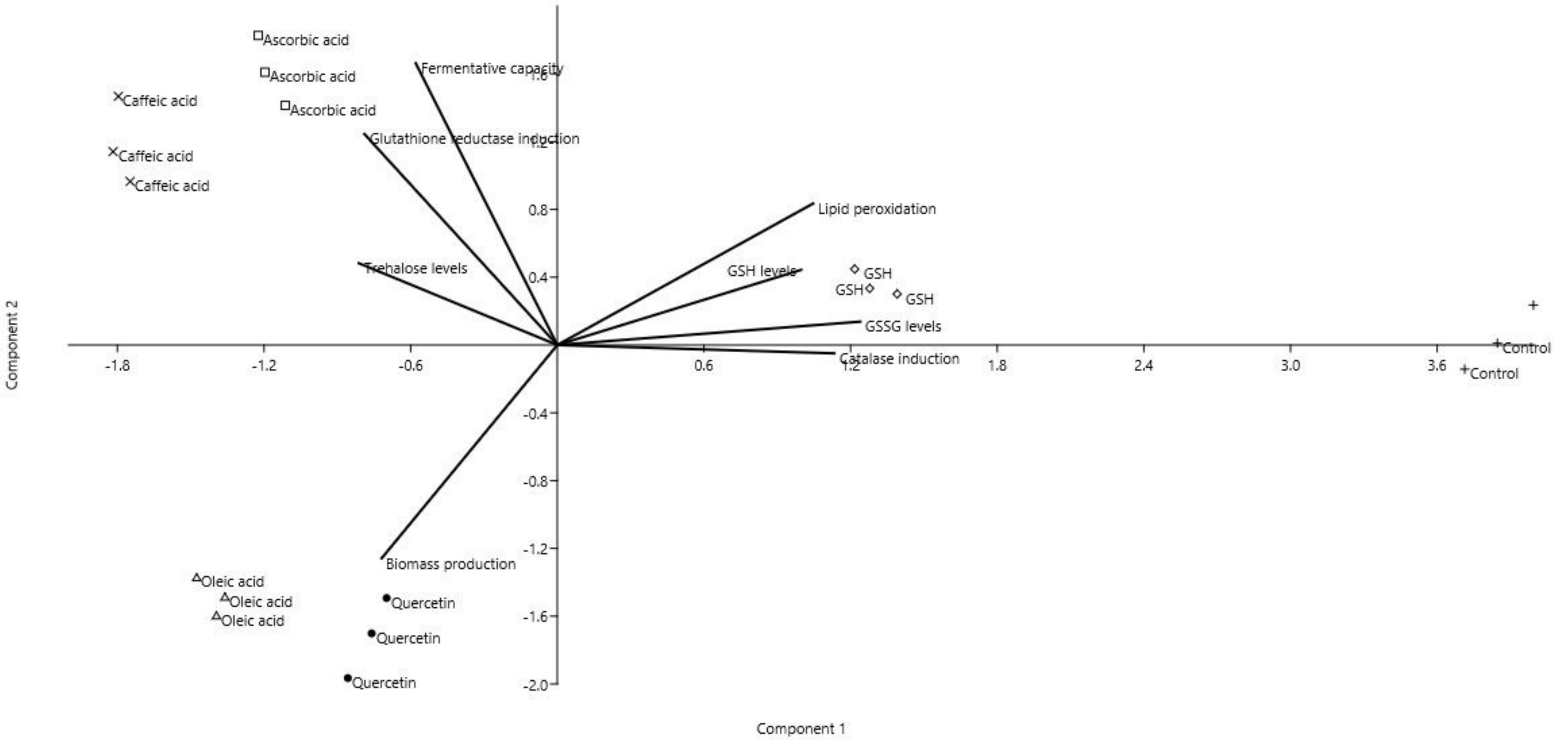
Ascorbic acid
Ascorbic acid
Ascorbic acid
Caffeic acid
Caffeic acid
Caffeic acid
Fermentative capacity
Glutathione reductase induction
Lipid peroxidation
Trehalose levels
GSH levels
GSH
GSH
GSH
GSSG levels
Catalase induction
Control
Control
Biomass production
Oleic acid
Oleic acid
Oleic acid
Quercetin
Quercetin
Quercetin
0.8
0.4
-0.4
-0.8
-1.2
-1.6
-2.0
-1.8
-1.2
-0.6
0.6
1.2
1.8
2.4
3.0
3.6
Component 1
Component 2

G) P6

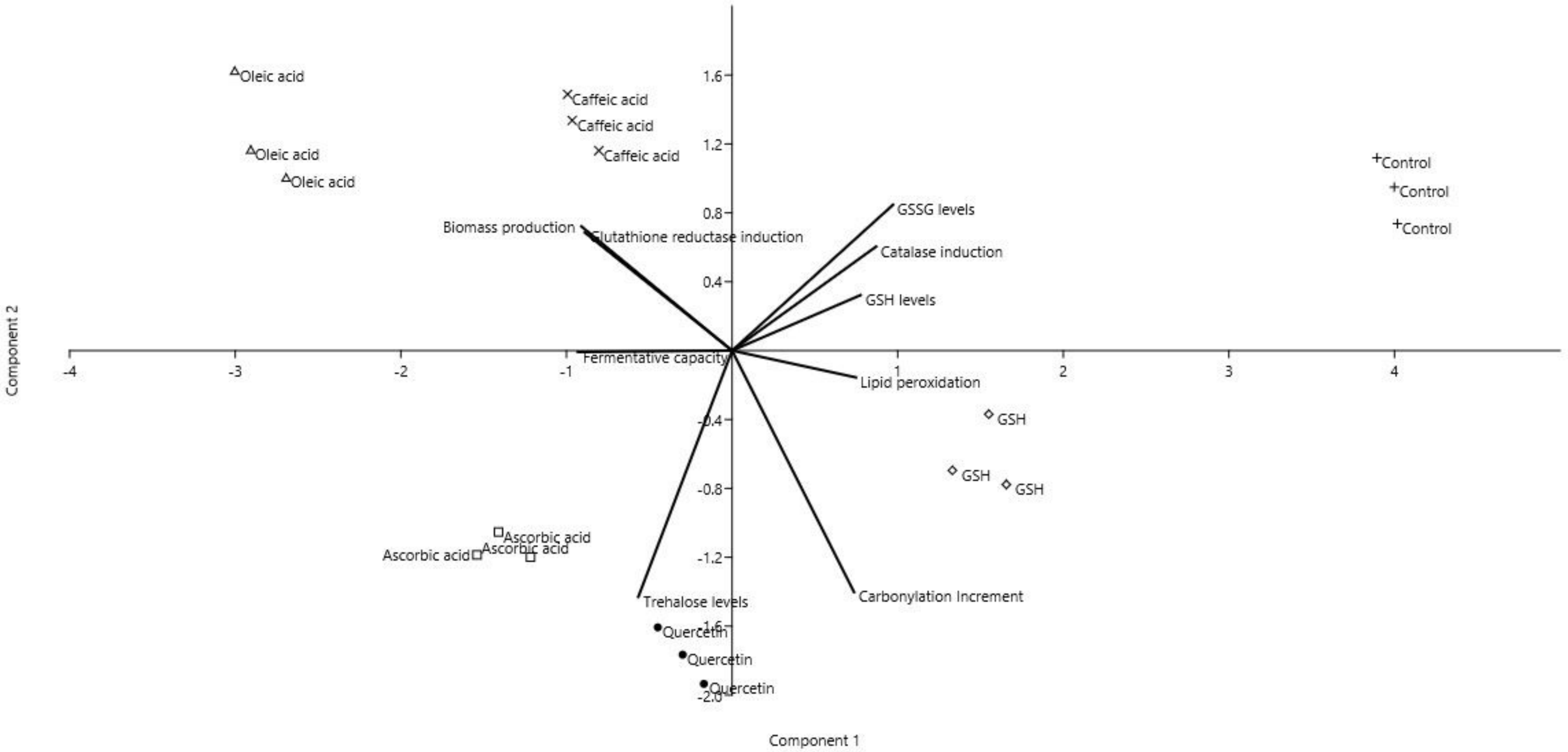

Oleic acid
Oleic acid
Oleic acid
Caffeic acid
Caffeic acid
Caffeic acid
Control
Control
Control
GSSG levels
Catalase induction
GSH levels
Biomass production
Glutathione reductase induction
Fermentative capacity
Lipid peroxidation
GSH
GSH
GSH
Ascorbic acid
Ascorbic acid
Ascorbic acid
Trehalose levels
Carbonylation Increment
Quercetin
Quercetin
Quercetin
1.6
1.2
0.8
0.4
-0.4
-0.8
-1.2
-1.6
-2.0
-4
-3
-2
-1
1
2
3
4
Component 1
Component 2

Fig. S1 Principal Components (PCA) statistical analysis of the antioxidant molecules effects on the physiological and biochemical biomarkers for each study strains with a total variance represented of 62%. Component 1 reflects an average 39% total variance (with a positive correlation with ascorbic acid and caffeic acid supplementations) and Component 2 reflects an average 23% total variance (with a positive correlation with oleic acid supplementation). The lines belong to variance of dependent variables or biochemical biomarkers measured (biomass yield, fermentative capacity, lipid peroxidation, protein carbonylation, protective metabolites and enzymatic activities) arranged in two dimensions according to the components 1 and 2. Treatment condition is labelled with different symbols: Control (+); Quercetin (●); Ascorbic acid (□); Caffeic acid (X); Oleic acid (Δ) and GSH (◊). (**A**) T73; (**B**) D18; (**C**) D170; (**D**) 301; (**E**) D128; (**F**) D272 and (**G**) P6.

Table S1. Technological properties and oxidation biomarkers of ADY from *Saccharomyces* wine strains under molasses supplementation with different antioxidants (Table 1 below)

	Parameter	Control	0.20 mg/mL Quercetin	5 mM GSH
**T73**	**Biomass (OD_{600})**	18.42 (± 0.2)	18.42 (± 0.2)	20.19 (± 2.1)
	**FC (mL CO_2/10^7cells)**	10.52 (± 0.3)	14.99 (± 0.6)	12.01 (± 2.2)
	**LP (pmol MDA/mg protein)**	26.85 (± 1.0)	24.10 (± 1.0)	25.85 (± 0.7)
	**GSH (nmol/mg cell)**	1.67 (± 0.01)	1.21 (± 0.06)	2.28 (± 0.01)
	**Trehalose (µg/mg cells)**	100.1 (± 9.7)	504.8 (± 3.1)	628.4 (± 22)
	**Δ GR (U/mg prot)**	-0.52 (± 0.3)	-0.34 (± 0.31)	0.11 (± 0.15)
	**Δ Catalase (U/mg prot)**	18.96 (± 0.1)	12.21 (± 0.28)	7.79 (± 0.90)
**D18**	**Biomass (OD_{600})**	16.64 (± 0.1)	22.94 (± 2.1)	15.81 (± 3.1)
	**FC (mL CO_2/10^7cells)**	8.39 (± 0.9)	7.55 (± 0.7)	7.23 (± 1.1)
	**LP (pmol MDA/mg protein)**	24.11 (± 2.3)	23.29 (± 1.3)	17.31 (± 0.1)
	**GSH (nmol/mg cell)**	0.91 (± 0.02)	1.31 (± 0.07)	2.20 (± 0.1)
	**Trehalose (µg/mg cells)**	193.5 (± 10)	175.4 (± 2.3)	83.3 (± 4.0)
	**Δ GR (U/mg prot)**	-0.17 (± 0.1)	-0.58 (± 0.27)	0.35 (± 0.23)
	**Δ Catalase (U/mg prot)**	31.26 (± 0.7)	6.16 (± 1.81)	5.36 (± 0.75)
**D170**	**Biomass (OD_{600})**	17.61 (± 0.2)	16.07 (± 0.6)	18.50 (±0.5)
	**FC (mL CO_2/10^7cells)**	17.18 (± 0.1)	9.35 (± 0.7)	17.26 (± 1.3)
	**LP (pmol MDA/mg protein)**	23.34 (± 0.9)	26.71 (± 0.7)	42.95 (± 0.4)
	**GSH (nmol/mg cell)**	1.24 (± 0.03)	1.18 (± 0.07)	1.18 (± 0.04)
	**Trehalose (µg/mg cells)**	155.5 (± 2.8)	166.6 (± 21)	80.9 (± 2.1)
	**Δ GR (U/mg prot)**	2.57 (± 0.7)	1.40 (± 0.13)	2.10 (± 0.30)
	**Δ Catalase (U/mg prot)**	0.28 (± 1.7)	4.07 (± 0.96)	6.58 (± 3.85)
**D301**	**Biomass (OD_{600})**	17.66 (± 0.4)	17.61 (±0.1)	13.97 (±0.1)
	**FC (mL CO_2/10^7cells)**	3.58 (± 0.1)	2.63 (± 1.2)	1.83 (± 0.1)
	**LP (pmol MDA/mg protein)**	20.08 (± 1.7)	26.92 (± 0.35)	55.34 (± 1.3)
	**GSH (nmol/mg cell)**	1.20 (± 0.05)	1.26 (± 0.11)	1.22 (± 0.01)
	**Trehalose (µg/mg cells)**	141.1 (± 22)	75.3(± 9.8)	15.3 (± 7.6)
	**Δ GR (U/mg prot)**	0.89 (± 0.23)	0.38 (± 0.93)	1.40 (± 0.23)
	**Δ Catalase (U/mg prot)**	67.76 (± 0.4)	27.79 (± 3.72)	15.64 (± 0.60)
**D128**	**Biomass (OD_{600})**	14.11 (± 0.1)	23.81 (± 0.1)	33.19 (± 2.6)
	**FC (mL CO_2/10^7cells)**	2.33 (± 0.6)	1.97 (± 0.1)	1.26 (± 0.6)
	**LP (pmol MDA/mg protein)**	22.00 (± 1.8)	11.32 (± 1.9)	22.00 (± 0.6)
	**GSH (nmol/mg cell)**	0.43 (± 0.05)	1.28 (± 0.13)	1.93 (± 0.29)
	**Trehalose (µg/mg cells)**	153.8 (± 11)	342.9 (± 1.0)	242.5 (± 2.3)
	**Δ GR (U/mg prot)**	1.70 (± 0.28)	1.60 (± 0.36)	1.40 (± 0.26)
	**Δ Catalase (U/mg prot)**	0.39 (± 1.1)	3.52 (± 0.41)	5.98 (± 0.75)
**D272**	**Biomass (OD_{600})**	11.26 (± 0.2)	22.13 (± 0.3)	5.57 (± 1.9)
	**FC (mL CO_2/10^7cells)**	10.42 (± 0.4)	5.36 (± 1.3)	12.15 (± 1.4)
	**LP (pmol MDA/mg protein)**	20.83 (± 1.4)	11.11 (± 1.8)	13.03 (± 0.2)
	**GSH (nmol/mg cell)**	2.42 (± 0.22)	1.13 (± 0.01)	3.04 (± 0.01)
	**Trehalose (µg/mg cells)**	190.9 (± 2.5)	518.9 (± 10)	386.9 (± 3.6)
	**Δ GR (U/mg prot)**	1.10 (± 0.20)	1.20 (± 0.05)	1.20 (± 0.06)
	**Δ Catalase (U/mg prot)**	-2.24 (± 0.3)	2.10 (± 0.36)	2.45 (± 0.01)
**P6**	**Biomass (OD_{600})**	13.72 (± 1.1)	24.71 (± 0.2)	24.6 (± 0.6)
	**FC (mL CO_2/10^7cells)**	12.53 (± 0.6)	15.07 (± 1.3)	11.65 (± 0.3)
	**LP (pmol MDA/mg protein)**	22.50 (± 0.9)	18.80 (± 1.4)	16.02 (± 0.7)
	**GSH (nmol/mg cell)**	3.65 (± 0.38)	1.22 (± 0.21)	4.23 (± 0.03)
	**Trehalose (µg/mg cells)**	252.6 (± 24)	784.1 (± 2.9)	629.7 (± 4.9)
	**Δ GR (U/mg prot)**	1.20 (± 0.01)	1.20 (± 0.08)	1.10 (± 0.14)
	**Δ Catalase (U/mg prot)**	0.064 (± 0.1)	0.91 (± 0.20)	0.80 (± 2.70)

FC (Fermentative capacity); LP (Lipid peroxidation), GSH (reduced glutathione) and GR (Glutathione reductase activity). Highlighting indicates significant difference respect to the control from non-supplemented molasses with $p < 0.05$. In brackets SD value from three independent experiments.
